# Dynamic Disintegration of Explosively-Driven Metal Cylinder with Internal V-Grooves

**DOI:** 10.3390/ma14030584

**Published:** 2021-01-27

**Authors:** Mingxue Zhou, Cheng Wu, Fengjiang An, Shasha Liao, Dongyu Xue, Jian Liu

**Affiliations:** State Key Laboratory of Explosion Science and Technology, Beijing Institute of Technology, Beijing 100081, China; zmxbit@163.com (M.Z.); anfengjiang@bit.edu.cn (F.A.); haha121@bit.edu.cn (S.L.); xuedychn@gmail.com (D.X.); 2120170237@bit.edu.cn (J.L.)

**Keywords:** cylindrical shell, internal V-grooves, explosive, fracture strain, failure mechanism

## Abstract

Machining V-shaped grooves to the internal surface of cylindrical shells is one of the most common technologies of controlled fragmentation for improving warhead lethality against targets. The fracture strain of grooved shells is a significant concern in warhead design. However, there is as yet no reasonable theory for predicting the fracture strain of a specific grooved shell; existing approaches are only able to predict this physical regularity of non-grooved shells. In this paper, through theoretical analysis and numerical simulations, a new model was established to study the fracture strain of explosively driven cylindrical shells with internal longitudinal V-grooves. The model was built based on an energy conservation equation in which the energy consumed to create a new fracture surface in non-grooved shells was provided by the elastic deformation energy stored in shells. We modified the energy approach so that it can be applicable to grooved shells by adding the elastic energy liberated for crack penetration and reducing the required fracture energy. Cylinders with different groove geometric parameters were explosively expanded to the point of disintegration to verify the proposed model. Theoretical predictions of fracture strain showed good agreement with experimental results, indicating that the model is suitable for predicting the fracture strain of explosively driven metal cylinders with internal V-grooves. In addition, this study provides an insight into the mechanism whereby geometric defects promote fracturing.

## 1. Introduction

The dynamic fragmentation of an explosively driven cylindrical shell is a highly complex phenomenon in which the shell is first accelerated outwards by an intense shock wave and highly pressurized detonation products, then undergoes large thermoplastic deformations, and eventually ruptures into discrete fragments with different velocities. The main physical regularities in this process can be sorted into the fracture strain, fragment size and number distribution, fragment velocity, and failure mechanism [1,2,3]. Among them, the fracture strain at the moment the warhead shell disintegrates into discrete fragments is of particular importance in the design of munitions and armaments. Numerous studies have focused on this topic since the Second World War.

In 1944, Taylor [4] proposed a model to predict the fracture strain of an expanding cylindrical shell based on the tensile strength of the material, the internal detonation gas pressure, and the wall thickness. In this model, radial cracks initiated on the external surface of the shell penetrate inwards as the hoop stresses in the cylinder are converted from compressive to tensile. The shell is considered to be completely ruptured when the internal gas pressure drops to the yield stress of the material. Hoggatt and Recht [5] further developed Taylor’s theory by incorporating the mechanism of shear fracture. They pointed out that radial fracture only has priority under low-order explosive loading, with shear fracture dominating under higher detonation loads. The shell ruptures when the pressure inside the shell becomes equal to the hoop tensile stress. Based on the statistical distribution of microvoids in metals, Feng et al. [6] proposed a damage model for warhead shells under internal explosive loadings. The damage model was then combined with constitutional equations to predict the fracture strain of warhead shells. These studies laid the foundation for the topic of the fracture strain of shells filled with high explosives, and have been extensively cited by subsequent researchers [7,8,9,10].

Unfortunately, the aforementioned models either ignore the dependence of the critical fracture strain on the strain rate (which was confirmed by the experiments of Singh et al. [10]) or include a large number of material parameters that must be determined experimentally in advance. As a result, these models may not be applicable to the dynamic fragmentation of shells with varied structures. Considering the effect of the strain rate on shell fragmentation, Al-Hassani and Johnson [11] extended Taylor’s model. They derived the following expression:

(1) $$\frac{r_{f}}{r_{0}}={\left[\frac{n\rho Tr_{0}\left(\gamma -1\right)}{2\sigma }\right]}^{1/n\gamma }{\overset{\cdot }{\varepsilon }}^{2/n\gamma }$$

where *r*_f_ is the fracture radius of the shell, *r*_0_ is the initial inner radius of the shell, $\dot{\varepsilon }$ is the strain rate, and *n* is the geometric parameter of the shell, with *n* = 3 for a sphere and *n* = 2 for a cylinder. σ, ρ, and *T* denote the flow stress, density, and wall thickness of the shell, respectively. γ is the adiabatic exponent of the detonation products. In this model, the fracture strain ε can be expressed as (*r*_f_ − *r*_0_)/*r*_0_, which increases as the strain rate increases or the wall thickness decreases. Based on an energy consideration, Ivanov [12] derived a function relating the strain rate to the fracture strain of a thin-wall shell filled with explosives. He pointed out that the energy consumed to generate a new fracture surface was provided by the elastic deformation energy stored in the shell. The shell can only break up when the strain and the strain rate satisfy a certain relationship, which can be expressed as

(2) $$\mu^{2}{\overset{\cdot }{\varepsilon }}^{2}\varepsilon \left[\frac{\varepsilon +2}{2}\right]+\overset{\cdot }{\varepsilon }\left(2\mu \varepsilon -\alpha \right)+\ln\left(\varepsilon +1\right)=0$$

where μ and α are material constants, ε is the fracture strain, and $\dot{\varepsilon }$ is the strain rate. Ivanov pointed out that the critical fracture strain of the shell first increases and then decreases as the strain rate increases. At a certain strain rate, the critical rupture strain attains a maximum value. This point of view regarding the relationship between the strain rate and fracture strain has been confirmed by other researchers [10,13,14].

Compared with the comprehensive understanding of the physical regularities determining the fracture strain of continuous shells without geometric defects, there has been little theoretical analysis on the fracture strains of grooved shells. Machining V-shaped grooves to the internal surface of cylindrical shells is one of the most common technologies of controlled fragmentation, allowing the warhead case to break into designed masses [15,16,17]. These precuts in warhead shells act to increase the local stress and promote the propagation and penetration of cracks through wall thickness, thereby leading to earlier disintegration than in a naturally fragmenting or a continuous shell. In addition, unlike a naturally fragmenting shell, cracks in a grooved shell originate from the groove tips instead of the inner or outer surface of the shell. Consequently, the fracture strain of internally grooved shells may differ significantly from that of naturally fragmenting shells.

To better understand the physics of controlled fragmentation, it is necessary to investigate the fracture strain of grooved shells. In the current work, the model of Ivanov [12] was modified to predict the fracture strain of grooved shells by adding the effect of geometric defects on fracture promotion. In this model, more elastic energy was available to promote crack propagation and less energy was necessary to generate a new fracture surface inside the grooved shell than those inside the non-grooved shells. The good agreement between the theoretical predictions and experimental results indicates the accuracy of the model in predicting the fracture strain of grooved warhead shells. This study also facilitates a better understanding of the difference between the fracture mechanism of grooved shells and that of naturally fragmenting shells.

## 2. Theoretical Analysis

Ivanov [12] showed that the fracture energy required to disintegrate a continuous shell was provided by the deformation energy stored in the shell. Later studies [10,13,14] showed that the proposed energy approach provides good predictions for the fracture strain of continuous shells fabricated from mild steel.

However, a recent study from Yang et al. [18] showed that the precut grooves on shells experience higher levels of local stress and effective strain than the surrounding matrix, thereby preserving more deformation energy for cracks to propagate and penetrate inside a grooved shell than inside a continuous shell. Additionally, the grooves shorten the path that cracks must penetrate, and so less fracture energy is required to break up a warhead shell. Therefore, the model proposed by Ivanov may not be applicable for predicting the fracture strain of grooved shells and should be modified accordingly.

Herein, we modified Ivanov’s theory by increasing the liberated energy provided for crack propagation and decreasing the required fracture energy. We first considered the stress concentration in the vicinity of the groove tips after the impact. After adding the stress concentration effect to the expression of the liberated energy, we reduced the energy required for the cracks to penetrate through the shell. Finally, we derived the relationship among the fracture strain, strain rate, and groove geometric parameters based on the aforementioned energy conservation approach.

### 2.1. Stress Concentration Effect

The machining of grooves to warhead shells produces discontinuities that are not present on naturally fragmenting shells. These discontinuities often act to increase the local stress when the stress wave obliquely collides at the groove tips. This phenomenon of locally high stress caused by geometric defects in a component (i.e., steps, grooves, holes) is called stress concentration. The stress concentration factor (SCF), which reflects the degree of local stress concentration, can be expressed as [19]

(3) $$K=\frac{\sigma^{\prime }}{\sigma }$$

where *K* is the SCF induced by the geometric defects, $\sigma^{\prime }$ is the maximum stress in the vicinity of the discontinuities after impact, and σ is the nominal stress, or the stress that would occur at the same position in the absence of discontinuities.

**Figure 1:**
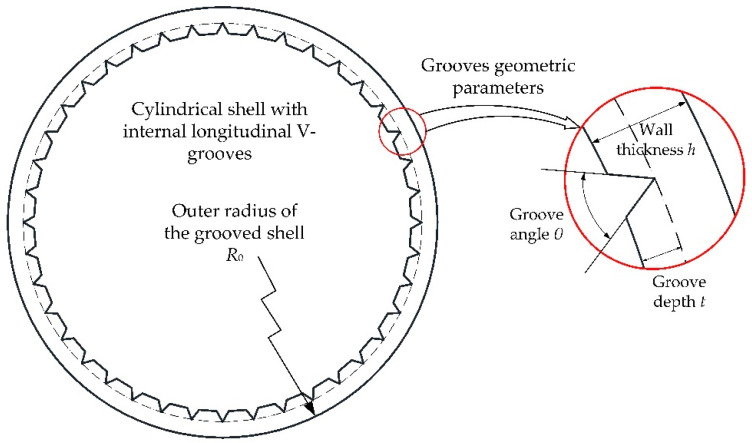
Cross-section of a cylindrical shell with V-grooves machined on the internal surface.

The main geometric parameters that may determine the SCF at the groove tips are the groove angle θ, the ratio of groove depth to wall thickness *t/h*, and the ratio of the initial outer radius of the shell to the wall thickness *R*_0_/*h*, as shown in Figure 1.

**Figure 2:**
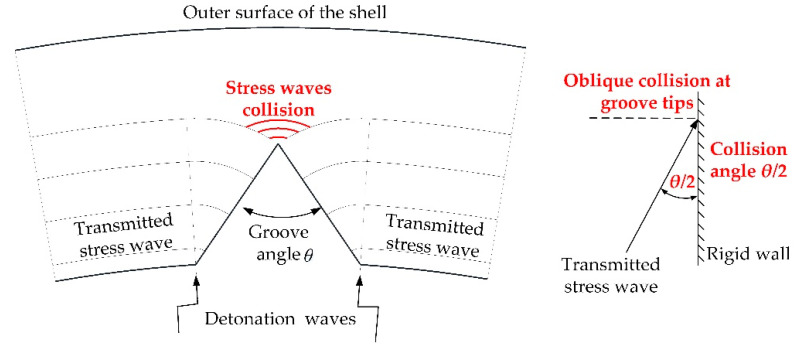
Collision of the transmitted waves at groove tips and oblique collision of the shock wave on the rigid wall.

First, after the detonation of explosives, the detonation wave propagates to the interface between the explosive and the grooved shell, whereupon the reflected wave propagates back to the detonation products and the transmitted stress wave propagates inwards to the grooved shell. When the transmitted waves reach the groove tips, they collide obliquely at a certain angle, leading to an overpressure compared to the surrounding matrix, as shown in Figure 2. This process can be simplified as the oblique collision of the shock wave on the rigid wall. According to the conventional oblique collision and irregular oblique collision (Mach collision) theory [20], the overpressure at the collision point is a function of the incident angle θ/2, and consequently the influence of the groove angle *θ* on the SCF cannot be ignored.

Second, according to the continuous case expansion model [5], the stress distribution is not uniform along the wall thickness of the shell. Recall that the SCF is the ratio of the maximum stress at the groove tips to the stress that would occur at the same position on the continuous shell, so the effect of *t*/*h* on the SCF should be considered.

Finally, as *R*_0_/*h* increases, the region adjacent to the groove tips gradually changes from a ring structure to a flat structure. The stress distribution inside the flat structure differs significantly from that inside the ring structure, so *R*_0_/*h* is also one of the factors that may affect the SCF.

It is very difficult to derive the expression of the SCF for problems with high strain rates and large deformations, such as explosion and hypervelocity impact. Herein, we made a simple analysis of the factors that may affect the SCF, and used $K(\theta ,t/h,R_{0}/h)$ to define the SCF at the groove tips. The specific expression of the SCF was obtained by numerical simulations and is described in detail in Section 3.

Due to the viscous drag mechanism of deformation, the yield point of soft steel can be assumed to be dependent on the true strain rate $\dot{\varepsilon }$ with a constant value of the dynamic viscosity η [12], that is,

(4) $${\sigma \;=\;\sigma }_{0}+\overset{\cdot }{\varepsilon }{\eta \;=\;\sigma }_{0}+v\eta /R$$

where σ_0_ is the static value of the flow stress, *v* is the expanding velocity of the shell after explosion, and *R* is the expanding external radius of the shell.

Substituting Equation (4) into Equation (3), we obtain the local high stress at the groove tips:

(5) $$\sigma^{\prime }=\;K(\theta ,t/h,R_{0}/h)\sigma =K(\theta ,t/h,R_{0}/h)(\sigma_{0}+v\eta /R)$$

### 2.2. Dynamic Destruction of Grooved Shell

The dynamic crack growth on the grooved shell and the stress threshold for this critical growth are somewhat complicated [21], so we do not go into the details of the crack development and final disintegration. Herein, we modified the theory from Ivanov by adding the liberated elastic energy provided for crack propagation and reducing the energy consumed to generate a new fracture surface. Some assumptions were introduced to simplify the derivation process.

**Figure 3:**
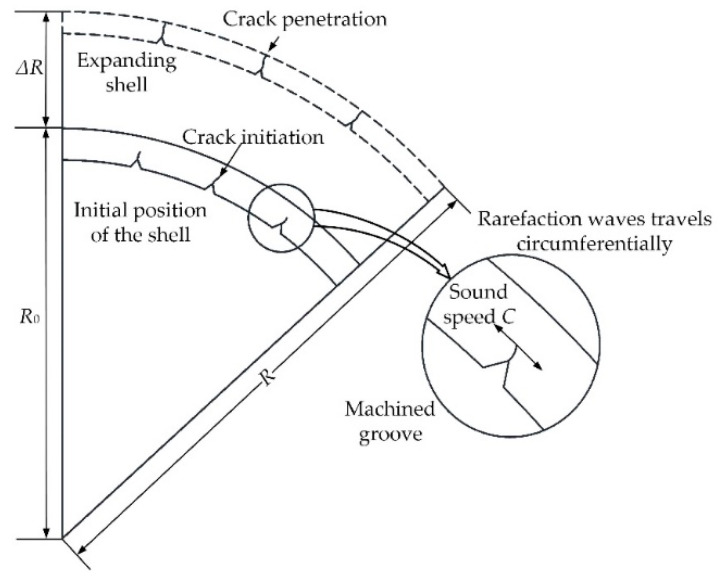
Crack propagation and expansion of a cylindrical shell subjected to internal explosive loading.

1. The shell geometry was assumed to be cylindrical, as presented in Figure 3. The cylinders were restricted to be thin-walled tubes with a relative wall thickness δ_0_ (*h*/*R*_0_) of no more than 1/8, so that the peripheral stress could be regarded as independent of the radius.

2. We studied a ring of unit width cut out from the central part of an infinite cylindrical shell and assumed that the detonation of explosives was instantaneous. Therefore, the problem could be simplified into a plane strain or two-dimensional problem.

3. The energy used for shell deformation was negligible relative to the kinetic energy of the shell, and the shell was pushed outwards by the high explosive loading in a small time interval. As a result, the expansion velocity v of the ring was assumed to be constant during the crack propagation process.

4. The material of the shell was assumed to be incompressible and the shape of the groove remained unchanged during the explosion.

Once the shell began to expand, cracks were assumed to initiate at the groove tips and propagate radially to the outer surface of the shell, whereupon the circumferential rarefaction wave unloaded the deformation area on the shell in two circumferential directions at the speed of sound *C* and released the elastic deformation energy to support crack propagation [12].

The width of the unloading zone on one side in the interval Δ*t* can be expressed as

(6) Δl=C×Δt

and the radial displacement of the shell can be expressed as

(7) ΔR=v×Δt

so we obtain dl=C×dR/v. We assumed that the elastic energy released per unit volume on the grooved shell was $q^{\prime }$, and denote the fracture energy consumed by disintegration per unit surface area was λ. Then,

(8) $$\int_{}^{V}q^{\prime }dV^{\prime }=\lambda S^{\prime }$$

where $S^{\prime }=1\delta R(1-t/h)$. According to the study of Pike et al. [16], geometric designs for the controlled fragmentation perform better when the groove spacing is approximately the same as the shell thickness *h*. Before the grooved shell is deformed, the volume of a single fragment can be expressed as $(h^{2}-t^{2}tg(\theta /2))\times \delta R$. The ratio of the volume of the fragment generated by the grooved shell to the volume of the fragment yielded by the intact shell can be expressed as $\left(1-t^{2}tg(\theta /2)/h^{2}\right)$. Then in the process of rarefaction wave propagation, the volume of the unloaded grooved shell can be expressed as- $dV^{\prime }=2\delta Rdl(1-t^{2}tg(\theta /2)/h^{2})$.

The elastic energy *q* per unit volume on the non-grooved shell can be written as [12]

(9) $$q=\sigma^{2}\left(1-\nu^{2}\right)/2E$$

where σ, *E*, and *ν* denote the hoop stress of plastic flow, Young’s modulus, and the Poisson ratio, respectively. For an incompressible shell material, we can set *ν* = 0.5.

Substituting Equation (5) into Equation (9), we obtain the elastic energy per unit volume on the grooved shell:

(10) $$q^{\prime }=K{\left(R_{0}/h,t/h,\theta \right)}^{2}\times \sigma^{2}\left(1-\nu^{2}\right)/2E$$

In Ivanov’s theory [12], the relationship between the elastic energy released and the energy consumed by the destruction of a naturally fragmenting shell is expressed as

(11) $$\int_{}^{V}qdV=\lambda S$$

where dV=2δRdl and S=1δR. Clearly, the effect of the groove geometry on shell destruction promotion was added to the theory proposed by Ivanov.

The material of the expanding shell was assumed to be incompressible, so that $\delta_{0}R_{0}^{2}\;=\;\delta R^{2}$. Thus, from Equation (8), we obtain

(12) $$\frac{2RC}{v\lambda (1-t/h)}(1-t^{2}tg\frac{\theta }{2}/h^{2})\int_{R_{0}}^{R}\frac{q^{\prime }dR}{R}=1$$

Substituting Equation (10) into Equation (12) and integrating yields

(13) $$\begin{array}{l} \frac{K{\left(\theta ,t/h,R_{0}/h\right)}^{2}}{\left(1-t/h\right)}(1-t^{2}tg\frac{\theta }{2}/h^{2})\left[\mu^{2}{\overset{\cdot }{\varepsilon }}^{2}\varepsilon (\varepsilon +2)/2+\ln(1+\varepsilon )\right]\\ +\overset{\cdot }{\varepsilon }\left[\frac{K{\left(\theta ,t/h,R_{0}/h\right)}^{2}2\mu \varepsilon }{\left(1-t/h\right)}(1-t^{2}tg\frac{\theta }{2}/h^{2})-\alpha \right]=0 \end{array}$$

where μ = η/σ_0_, and $\alpha =4\lambda E/3C\sigma_{0}^{2}$. From [12], μ = 0.85 × 10^−4^ s and α = 1.67 × 10^−4^ s were good estimations for the thin-walled tubes fabricated from soft steels, such as St. 3, St. 20, and St. 35.

Equation (13) completely determines the dynamic destruction of a grooved shell subjected to internal explosive loadings. The problem addressed in Section 3 is how to derive expressions for $K(\theta ,t/h,R_{0}/h)$.

## 3. Numerical Simulation

Although extensive analytical and experimental studies focused on the determination of the SCFs of cylindrical shells, plates, and round bars with periodic notches or grooves [22,23,24], most were restricted to static or quasi-static scenarios. Such approaches may not be able to determine the SCFs of materials that undergo large deformations and high strain rate, such as warhead explosions and hypervelocity impacts. Fortunately, computational methods are effective in evaluating the SCFs under impulsive loading conditions [15,18,19,25]. Therefore, in this section, the effects of the groove parameters on the SCFs were explored through numerical simulations using the ANSYS/LS-DYNA software package, which is widely used to handle non-linear dynamics problems.

### 3.1. Numerical Simulation Models

**Figure 4:**
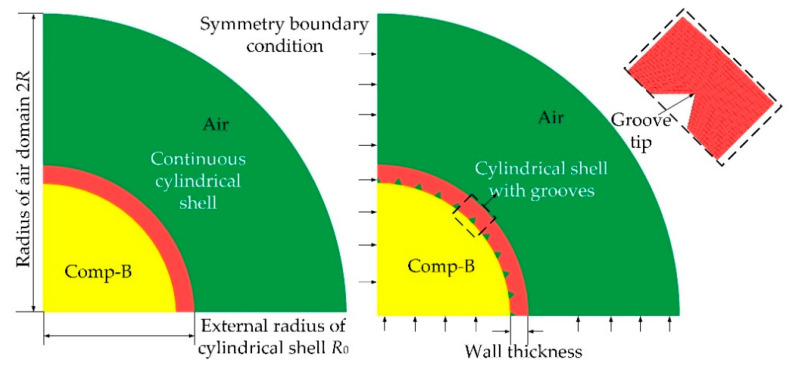
Simulation model of cylindrical shells with discontinuity and no discontinuity under internal explosive loading.

Three-dimensional (3D) models with hexahedral meshes (solid 164) and a coupled Arbitrary Lagrange–Eulerian (ALE) algorithm were established to calculate the SCFs at the groove tips, as shown in Figure 4. The metallic cylinders were modeled with Lagrange meshes, whereas the explosive charge and the air were modeled with Eulerian meshes. Note that only a single-layer solid grid was applied in the numerical models. It seems that the structure was in a plain-stress state rather than a plane-strain state since the simulation model was very thin in the normal direction or in the Z direction. However, we studied the ring of unit width cut out from the central part of an infinite cylindrical shell, and we constrained the grid displacement of the ring in the normal direction or in the Z direction. As a result, the displacement or the strain of the ring in the Z direction was zero and the structure could be treated as a plane-strain problem (the stress in the Z direction was not zero). The method of dealing with boundary conditions of the simulation models was consistent with the work of Yang et al. [18], who studied the effects of pre-notches on the self-organization of multiple adiabatic shear bands (ASBs) in a long 7075 aluminum alloy cylinder by means of the finite element method (FEM), and a plane-strain state was obtained. To avoid pressure reflection, a “flow out” boundary condition was applied to the border of the air grid. On the grounds of symmetry, only a quarter of the shell was included in the model. The radial dimension of the air domain was set to twice the outer radius of the shell, which was sufficient for the shell to break into discrete fragments. As a trade-off between accuracy and computational efficiency, we chose a mesh size of 1/50 of the shell thickness for both the shell and the air. The determination of the mesh size and a detailed mesh sensitivity analysis are presented in Section 3.2.

The material model *MAT_PIECEWISE_LINEAR_PLASTICITY (MAT_024) was used to describe the elastic–plastic response of a grooved shell fabricated from AISI 1020 steel under explosion loading. The effect of the strain rate on the material properties was considered based on the Cowper–Symonds model [26],

(14) $${\sigma \;=\;\sigma }_{0}\left[1\;+\;{\left(\overset{\cdot }{\varepsilon }/C\right)}^{\frac{1}{P}}\right]$$

where *σ* is the flow stress of the material and $\dot{\varepsilon }$ is the strain rate. *P* and *C* are related to the strain rate, with *P* = 40 and *C* = 5 providing a good estimation for mild steels [26]. σ_0_ is the static value of the flow stress.

The standard Jones–Wilkins–Lee (JWL) equation of state [27] was employed to define the burn and flow of the explosive Comp-B, namely,

(15) $$P=C_{1}\left(1-\frac{\omega }{r_{1}\nu }\right)e^{-r_{1}\nu }+C_{2}\left(1-\frac{\omega }{r_{2}\nu }\right)e^{-r_{2}\nu }+\frac{\omega E}{\nu }$$

where *P* is the detonation pressure, *E* is the internal energy per initial volume, and ν is the initial relative volume. The other coefficients are material constants. The air is described by the ideal gas equation of state (EOS). The material properties used for the steel and the explosive are taken from literatures [26,28], respectively.

### 3.2. Mesh-Sensitivity Analysis

**Figure 5:**
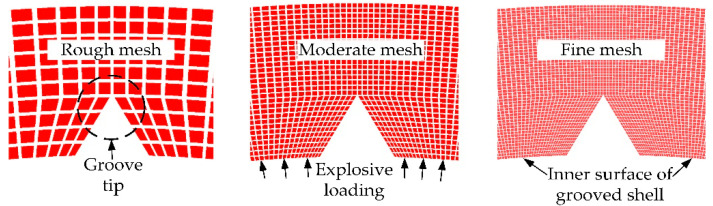
Detailed view of cylindrical shells with different mesh resolutions. Mesh densities at groove tips were increased for stress concentration analysis.

The case with *R_0_* = 50 mm, *h* = 6 mm, *t* = 2.4 mm, and θ = 60 degrees was selected to explore the effect of the mesh resolution on the numerical results. The mesh sizes were classified into several groups: (a) rough meshes 1/10 of the shell thickness, (b) moderate meshes 1/30 of the shell thickness, and (c) fine meshes 1/50 of the shell thickness, as shown in Figure 5. The mesh density of continuous shells was set to be identical to that of the corresponding grooved shells. The SCFs were defined as the ratio of the maximum circumferential stress at the groove tips after impact to the stress that would occur at the same position inside the continuous shells.

**Figure 6:**
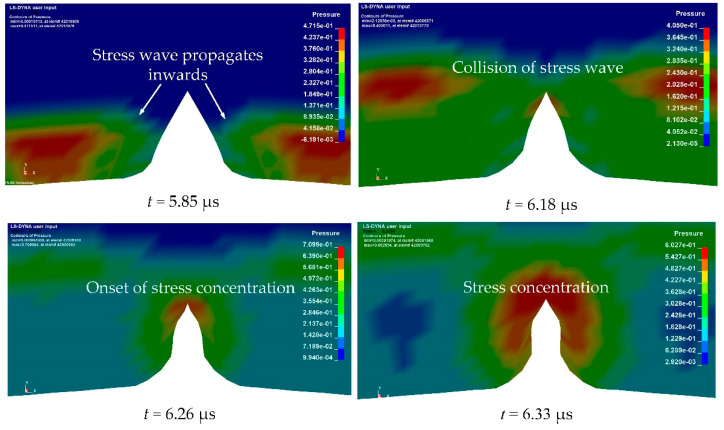
Numerical observations of the stress wave propagation and onset of stress concentration inside the grooved shell with *R*_0_ = 50 mm, *h* = 6 mm, *t* = 2.4 mm, and θ = 60 degrees at 5.85, 6.18, 6.26, and 6.33 μs after detonation.

Figure 6 illustrates the propagation of the stress wave and the onset of stress concentration inside a grooved shell. It is evident that the stress distribution inside the shell was non-uniform and that stress concentration occurred at groove tips. After detonation of the explosive, the detonation front propagated to the interface between the shell and the explosive charge, whereupon the transmitted stress waves continued to propagate inwards. When the transmitted stress waves collided at the groove tips, the region in the vicinity of the groove experienced a higher level of local stress than the steel matrices and stress concentration occurred.

**Figure 7:**
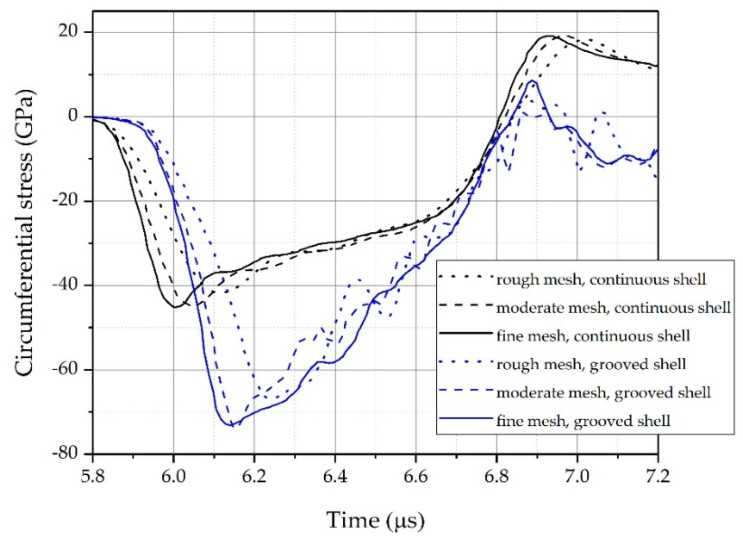
Circumferential stress as a function of time after impact for each mesh-sensitivity trial.

Figure 7 presents the circumferential stress at the groove tips and the stress that would occur at the same position in a continuous shell. Clearly, due to the presence of the grooves, the circumferential stress at the groove tips was obviously higher than that at the continuous shell. For the current case, the SCF at the groove tips was around 1.62. Yang et al. [18] reported that the stress concentration caused by the geometric defects induced a higher level of local effective strain at groove tips than in the matrix. Feng and Bassim [29] found that the geometric defects enhanced the formation of shear bands in specimens. The shear bands in grooved specimens can get more energy to propagate than in the non-grooved shell. The mathematical model from Wright and Walter [30] suggested that the stress collapse of materials depends on the initial size of geometric defects. These results may indicate an earlier disintegration of a grooved shell than that of a continuous shell without geometric defects (grooves).

In addition, it was found that the simulation results were highly dependent on the mesh density. As the mesh density increased, the circumferential stress curves became steeper at the rising stage, and the peak stress increased. However, there were no evident differences between the circumferential stress curves using the moderate and fine meshes. The fine mesh was sufficient to guarantee the reliability and robustness of the current model. Thus, as a compromise between accuracy and computational efficiency, we used the fine mesh to discretize all simulation models.

### 3.3. Determination of SCFs

In terms of a cylindrical metallic shell machined with internal grooves, the groove angle θ, the ratio of groove depth to wall thickness *t*/*h*, and the ratio of shell outer radius to wall thickness *R*_0_/*h* were all possible factors that affected SCFs at groove tips. In this section, we report the results of a series of simulations conducted to explore the effect of these geometric parameters on the SCFs. The serial numbers of the simulations correspond exactly to the serial numbers in Table 1. Simulations 1, 2, and 3 were combined into component 1 to study the effect of θ on SCFs at groove tips. In each individual trial, *t*/*h* and *R*_0_/*h* were fixed and θ was changed. Then we compared simulations 1, 2, and 3 to study the influence of changes in *R*_0_/*h* and *t*/*h* on the SCFs curves with θ as the independent variable. Similarly, the effects of *t*/*h* and *R*_0_/*h* on SCFs were explored by component 2 (simulations 4, 5, and 6) and component 3 (simulations 7, 8, and 9), respectively.

**Figure 8:**
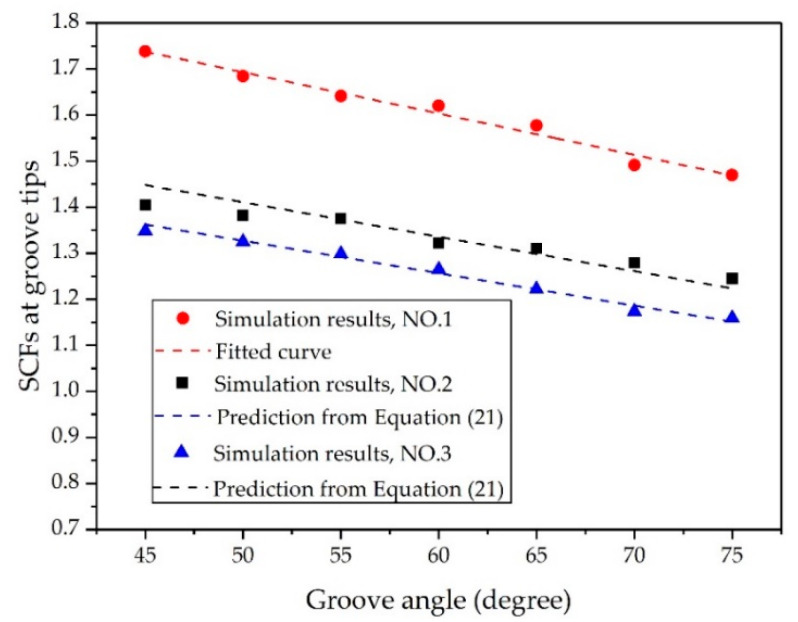
Relationship between groove angle θ and SCFs at groove tips.

**Figure 9:**
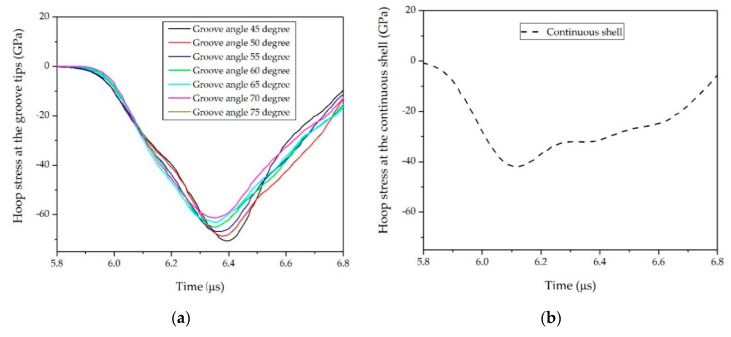
Hoop stress at groove tips and the stress at the same position inside the continuous shell (NO. 1): (**a**) relationship between groove angle θ and hoop stress at groove tips and (**b**) relationship between groove angle θ and the stress at same position inside the continuous shell.

We first considered the effects of θ. In Case NO. 1, NO. 2, and NO. 3, values of θ = 45°, 50°, 55°, 60°, 65°, 70°, and 75° were simulated to determine the influence on the SCFs, as shown in Table 1. The SCFs obtained with different θ are plotted in Figure 8. It was obvious that SCFs decreased as θ increased. This decline could be attributed to the change in the collision angle θ/2 of the transmitted stress waves. As shown in Figure 9, the absolute value of the peak hoop stress at groove tips induced by the oblique collision of the transmitted stress waves decreased as the groove angle increased, whereas the peak hoop stress at the same position inside the continuous shell remained unchanged. Therefore, the increase in groove angle θ led to a decrease in the SCFs at groove tips. This result was similar to the earlier observations from Li [31], who found that the collision pressure of detonation waves presents an almost linear decrease as the incident angles increase.

**Figure 10:**
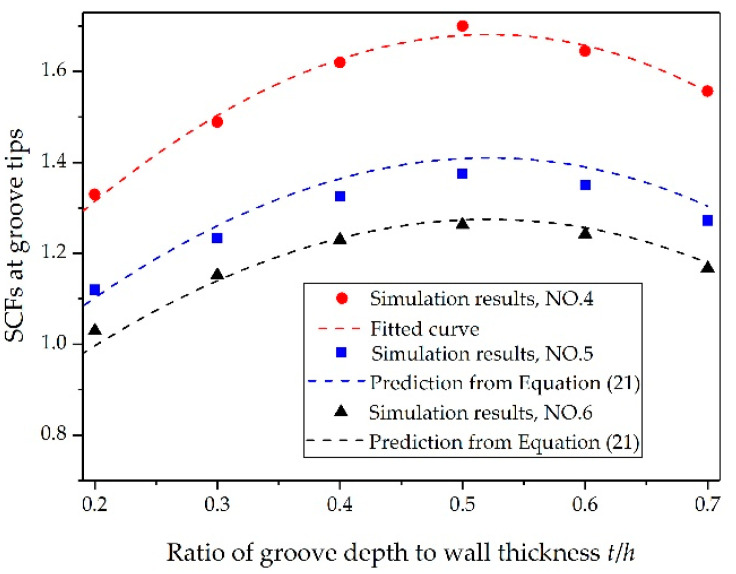
Relationship between *t*/*h* and SCFs at groove tips.

**Figure 11:**
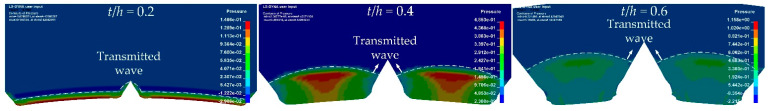
Propagation of the transmitted stress waves along the inclined groove wall.

**Figure 12:**
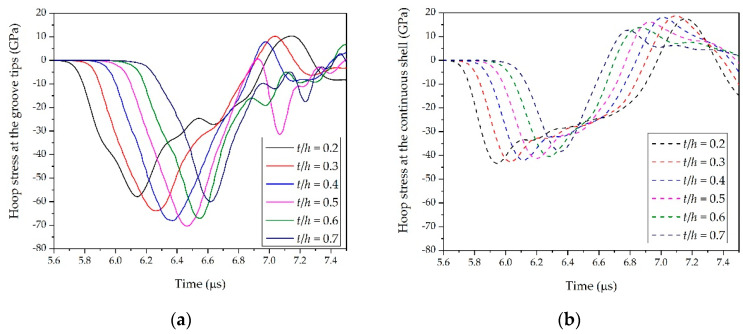
Hoop stress at groove tips and the stress at the same position inside continuous shell (NO. 4): (**a**) relationship between *t*/*h* and hoop stress at groove tips and (**b**) relationship between *t*/*h* and the stress at the same position inside the continuous shell.

Subsequently, we considered groove depths of *t/h* = 0.2, 0.3, 0.4, 0.5, 0.6, and 0.7 to investigate the effects of *t/h* on the SCFs; the other parameters were listed in Table 1. As shown in Figure 10, the SCFs first increased as *t*/*h* grew, and then decreased when *t*/*h* continued to grow. Figure 11 shows propagation of the transmitted stress waves along the inclined groove wall. As *t*/*h* increased, the collision angle of the two transmitted waves gradually decreased to θ/2. According to the aforementioned results, the absolute value of the peak hoop stress at the collision point would increase as the collision angle decreased (see Figure 9a). However, when *t*/*h* continued to increase, the collision angle of the transmitted wave remained unchanged, whereas the amplitude of the transmitted wave inside the continuous shell decreased as *t*/*h* increased (see Figure 12b). The absolute value of the peak hoop stress at groove tips is determined by both the amplitude of transmitted stress wave and the collision angle. Therefore, the absolute value of the peak hoop stress and the SCFs at the tip of the groove would first increase and then decrease (see Figure 10 and Figure 12a). This tendency was consistent with the finding of Kosmatka [28], who computed the SCFs of warhead cases with a series of groove depths from an MSC/NASTRAN code.

**Figure 13:**
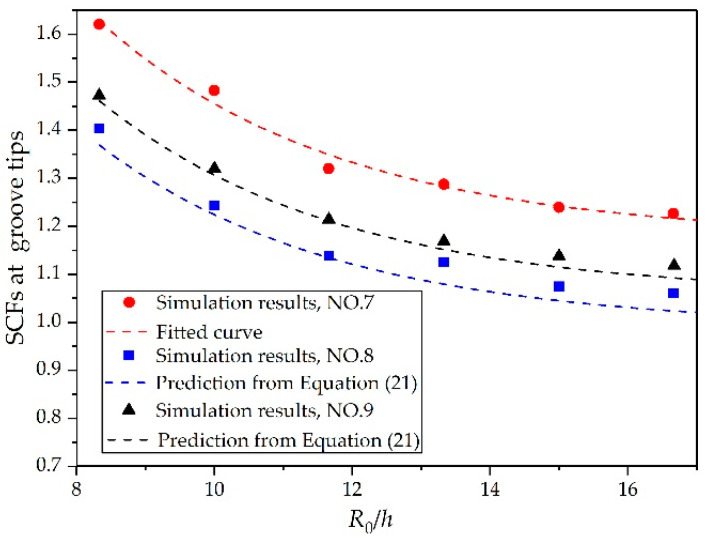
Relationship between *R*_0_/*h* and SCFs at groove tips.

**Figure 14:**
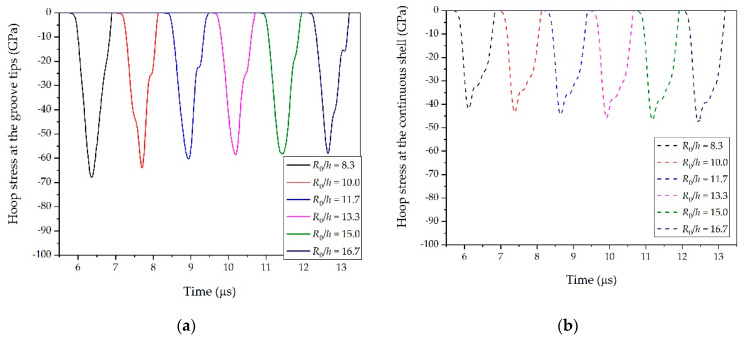
Hoop stress at groove tips and the stress at the same position inside the continuous shell (NO. 7): (**a**) relationship between *R*_0_/*h* and hoop stress at groove tips and (**b**) relationship between *R*_0_/*h* and the stress at the same position inside the continuous shell.

The *R*_0_/*h* was also a possible factor that could determine SCFs. Having analyzed the effects of *t*/*h* and θ, we simulated cylindrical shells with *R*_0_/*h* = 16.7, 15, 13.3, 11.6, 10, and 8.3 to investigate the effects on the SCFs. The results are illustrated in Figure 13. The amplitude of the stress wave at the groove tips presented a polynomial decrease as *R*_0_/*h* increased, whereas the amplitude of the stress wave at the same position inside the continuous shell increased linearly as *R*_0_/*h* increased (see Figure 14). As a result, the SCFs at the groove tips decreased continuously as the region in the vicinity of groove tips changed gradually from a ring structure to a flat structure.

It can be seen from Figure 8, Figure 10, and Figure 13 that the curves in each figure originated from one master curve multiplied by a magnification factor. Therefore the factorization was reasonable. In other words, the effects of θ, *t*/*h*, and *R*_0_/*h* on SCFs were not mutually coupled, but rather independent of each other. As a result, the expression for the SCFs at groove tips can be expressed as

(16) $$K\left(\theta ,t/h,R_{0}/h\right)=g\left(\theta \right)\times \omega \left(t/h\right)\times \varphi \left(R_{0}/h\right)$$

where g(θ), ω(t/h), and $\varphi \left(R_{0}/h\right)$ represent the effects of θ, *t*/*h*, and *R*_0_/*h*, respectively.

The SCFs exhibited a linear decline as θ increased. Therefore, a linear function was used to fit the data (simulation 1, R-Square = 0.98), namely,

(17) K(θ,0.4,8.33)=g(θ)×ω(0.4)×φ(8.33)=2.1411−0.00897θ

The SCFs first increased as *t*/*h* grew, and then decreased when *t*/*h* continued to grow. So we used a polynomial function to fit the simulation data, which can be expressed as (simulation 4, R-Square = 0.99)

(18) $$\begin{array}{l} K(60,t/h,8.33)=g(60)\times \omega (t/h)\times \varphi (8.33)=0.759\;+\;3.32(t/h)-\\ 2.56{\left(t/h\right)}^{2}-0.8{\left(t/h\right)}^{3} \end{array}$$

Again, we fitted the data for *R*_0_/*h* using the function (simulation 7, R-Square = 0.99)

(19) $$K(60,0.4,R_{0}/h)=g(60)\times \omega (0.4)\times \varphi (R_{0}/h)=1.175+4.968e^{\frac{-R_{0}/h}{3.475}}$$

Equations (17)–(19) can be combined to obtain

(20) $$\begin{array}{l} K(\theta ,0.4,8.33)\times K(60,t/h,8.33)\times K(60,0.4,R_{0}/h)=g\left(\theta \right)\times \omega (t/h)\times \varphi (R_{0}/h)\\ \times {\left[g\left(60\right)\times \omega (0.4)\times \varphi (8.33)\right]}^{2}=K(\theta ,t/h,R_{0}/h)\times K{\left(60,0.4,8.33\right)}^{2} \end{array}$$

Substituting Equations (17)–(19) into Equation (20) and simplifying yields

(21) $$\begin{array}{l} K\left(\theta ,t/h,R_{0}/h\right)=\frac{1}{2.57}\left[2.1411-0.00897\theta \right]\times \left[1.175+4.968e^{\frac{-R_{0}/h}{3.475}}\right]\\ \times \left[0.759\;+\;3.32(t/h)-2.56{\left(t/h\right)}^{2}-0.8{\left(t/h\right)}^{3}\right] \end{array}$$

which determines the SCFs at the groove tips.

**Figure 15:**
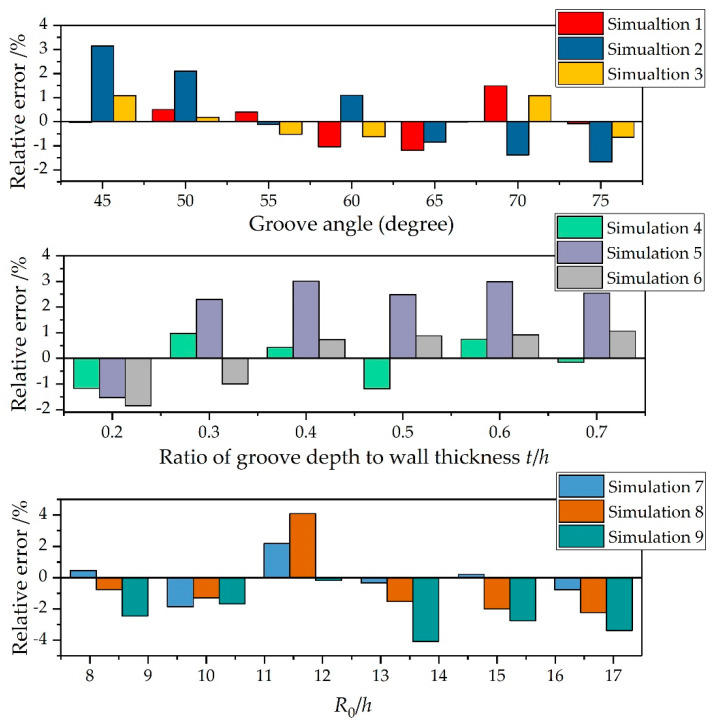
The relative error between the predictions from Equation (21) and the simulation results.

To further validate the reliability and applicability of the present formula, we made comparisons between the predictions from Equation (21) and the simulation results from Figure 8, Figure 10, and Figure 13. Figure 15 shows the relative error between the predictions from Equation (21) and the simulation results. Since Equation (21) was obtained by fitting the data of numerical simulations 1, 4, and 7, the maximum relative errors between Equation (21) and simulations 1, 4, and 7 in their respective components were the smallest. The maximum relative errors between Equation (21) and simulations 1, 4, and 7 were 1.49%, −1.19%, and 2.17%, respectively. The predictions from Equation (21) were also in good overall agreement with the other simulation results. For Component 1 (simulations 1, 2, and 3), the maximum relative error was 3.15% and the average relative error was 0.14%. For Component 2 (simulations 4, 5, and 6), the maximum relative error was 3.0% and the average relative error was 0.68%. For Component 3 (simulations 7, 8, and 9), the maximum relative error was 4.1% and the average relative error was −1.02%. These results may indicate that the established formula is applicable to determine the SCFs of warhead shells with internal grooves under internal explosive loadings.

This section determined the expressions for the SCFs $K(\theta ,t/h,R_{0}/h)$. By substituting Equation (21) into Equation (13), it was available to predict the fracture strain of warhead shells with specific geometric defects. The next section, therefore, moves on to validate the accuracy of the revised model discussed in Section 2.

## 4. Validation of the Proposed Model

This section reports the results of experiments to investigate the disintegration of explosively driven cylindrical shells with varied groove depths. The fracture strains were used to validate the applicability of the proposed formula.

### 4.1. Description of Experiment

**Figure 16:**
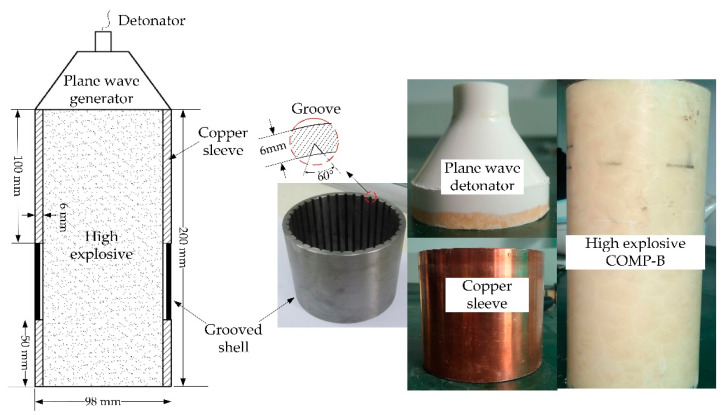
Schematic of experimental assembly. The grooved shell was placed between two copper sleeves and subjected to a plane-strain load that passed down the stacked cylinders.

As mentioned previously in Section 2, theoretical analysis of the dynamic destruction of grooved shells was based on a plane-strain assumption. That is, the explosively driven grooved shell was subjected to a pure radial load or an identical plane state in the longitudinal direction. To apply the plane-strain load to the grooved shell, an experiment assembly was designed, as illustrated in Figure 16. A plane wave generator was initialed at one end to propagate a near-planar detonation along the high explosive charge. The stacked cylinders, comprising two copper sleeves and a grooved shell, were filled with high explosive Comp-B and used to confine the explosive as the detonation wave passed down. Previous studies [8,28] showed that the rarefaction waves originating from the initiation end have a maximum axial propagation distance of twice the charge radius; the rarefaction waves originating from the opposite end have a maximum axial propagation distance equal to the charge radius. The grooved shell was located between the two copper sleeves so it was unaffected by the rarefaction waves originating from either end.

Shells with internal longitudinal V-grooves were fabricated from the GB/JB 20 steel (corresponding to the AISI 1020). The material is a medium carbon steel; its chemical constituents and mechanical properties can be obtained from the literature [32]. The grooved cylinders were constructed with an outer diameter of 98 mm, a wall thickness of 6 mm, and a length of 50 mm. The Comp-B explosive charge (diameter 86 mm, length 200 mm) was used to drive the shells outwards, which had a density of 1.68 g/cm^3^ and a detonation velocity of 7840 m/s. To investigate the effect of geometric defects on fracture promotion and for comparison with the theoretical prediction, cylindrical shells with different groove depths were designed and machined. The ratio of groove depth to wall thickness *t*/*h* ranged from 0.2–0.7. The angle of the symmetrical V-groove was set to 60°, and the groove spacing was set to be approximately equal to the wall thickness of 6 mm. The experimental conditions are summarized in Table 2.

**Figure 17:**
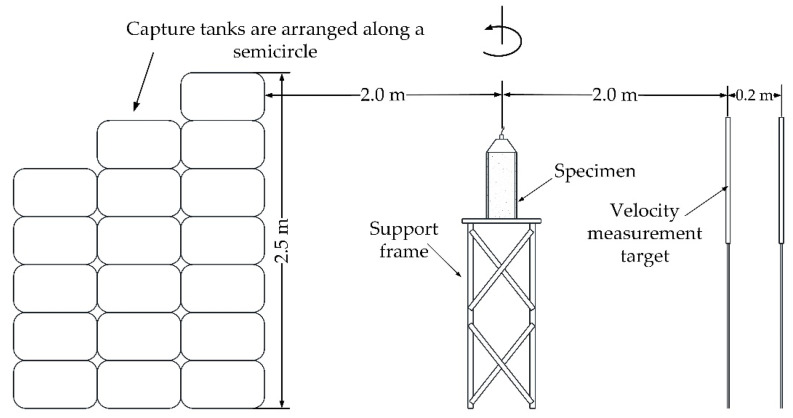
Elevation view of the experimental setup.

Figure 17 shows the experimental setup. Capture tanks filled with watered sawdust were used to collect the fragments generated by the explosively driven grooved shells. As the focus of this article is not the fragment mass and number distribution, we did not need to collect all the fragments and we arranged capture tanks along a semicircle centered on the experimental assembly with a radius of 2 m. The arrangement of the capture tanks was sufficient to retard enough fragments for examination and measurement. The fragment velocity was measured by two velocity measurement targets, which were placed 2 m away from the experimental assembly. The velocity measurement target, made up of two sheets of aluminum foil and an insulating paperboard, was connected to an oscilloscope. When fragments penetrated the velocity measurement target, a circuit was connected and an electrical signal was recorded by the oscilloscope. The fragment velocity *v* was then calculated from the time signal interval Δ*t* and corresponding displacement Δ*x* of the two measurement targets, namely, *v* = Δ*x*/Δ*t*.

### 4.2. Experiment Results

**Figure 18:**
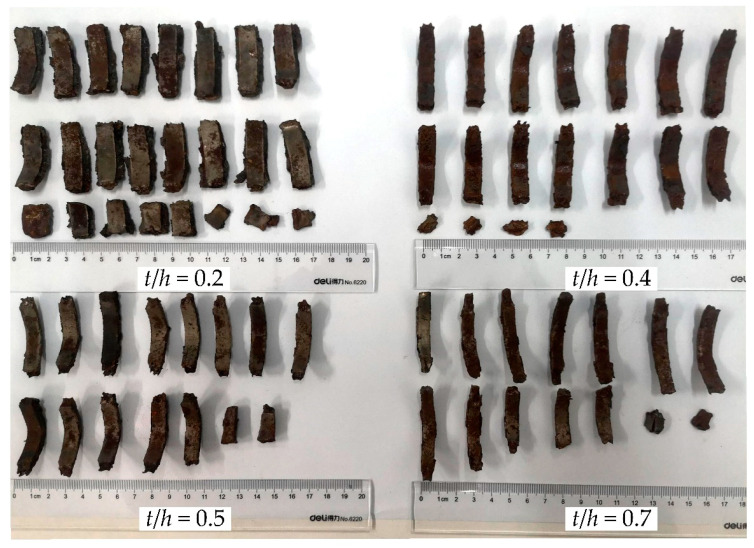
Recovered fragments generated from the grooved shell with varied ratios of groove depth *t* to wall thickness *h*.

After the explosion, recovered fragments exhibiting clear inner and outer surfaces were cleaned, dried, and sorted, as shown in Figure 18. It was obvious that the fragments underwent remarkable deformations, especially those from shells with a lower ratio of groove depth to wall thickness *t*/*h*. This may indicate that the radial compressive stress extruded the material, thinning the shell under the drive of explosive loading. However, at the onset of rupture, the deformation region inside the shell is rapidly unloaded by the release wave that propagates through the shell, and fragment deformation terminates [1,3,15]. As a result, the measured wall thickness of the fragments should coincide with that at the fracture moment, and the fracture strain of the grooved shells can be determined by the change in wall thickness [33], that is,

(22) $$\varepsilon_{f}=\frac{h_{0}-h_{f}}{h_{0}}=\frac{R_{f}-R_{0}}{R_{0}}$$

where ε_f_ is the fracture strain of grooved shells, *h*_0_ is the initial wall thickness, *h*_f_ is the average wall thickness of fragments, *R*_0_ is the initial outer radius of the shell, and *R*_f_ is the radius where shell ruptures.

The circumferential strain rate at the fracture time can be expressed as

(23) $$\overset{\cdot }{\varepsilon_{f}}=\frac{v}{R_{f}}$$

where $\overset{\cdot }{\varepsilon_{f}}$ is the strain rate of grooved shells at the fracture time and *v* is the initial fragment velocity. The fragment velocity, strain rate, and corresponding fracture strain measured in the experiments are listed in Table 3.

**Figure 19:**
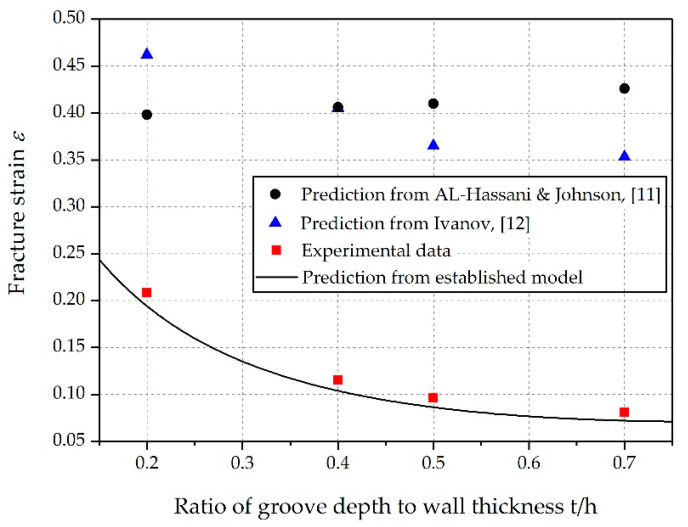
Fracture strain of explosively driven cylindrical shells with varied groove depths.

**Figure 20:**
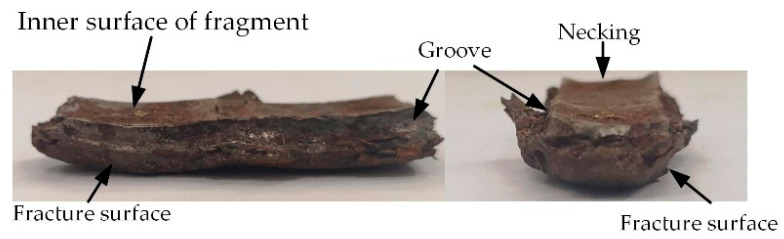
Necking and fracture surface of the recovered fragment.

The fracture strain predicted by the proposed model, along with the experimental data and results calculated by other formulas, are summarized in Table 3 and Figure 19. It can be clearly seen that the proposed model provided a reasonably more accurate estimate compared to other formulas. Note that the maximum error between the theoretical prediction and the experimental data regarding the fracture strain increased as *t*/*h* grew. There are several possible explanations for this result. First, the measurement error might be enlarged when the fracture strain is relatively small. Second, in the process of shell expansion, a small deformation occurs in the inner surface of fragments, which is similar to the necking phenomenon in material tensile tests (see Figure 20). This local necking thins the shell and might introduce additional errors to the measurements.

Despite these errors, there was an overall good agreement between the newly established model and the experimental data, indicating that our model can be applied to predict the fracture strain of the explosively driven cylindrical shells with internal V-grooves.

### 4.3. Discussion

Having validated the accuracy of the proposed model, we now address the mechanism of geometric defects on fracture promotion. As mentioned in Section 2, we modified Ivanov’s theory to predict the destruction of grooved shells by adding the elastic energy per unit volume liberated for crack penetration and reducing the total fracture energy. To clarify the complex nature of this energy approach, we planned to figure out how the changes in the elastic energy and fracture energy induced by geometric defects promote fracturing.

**Figure 21:**
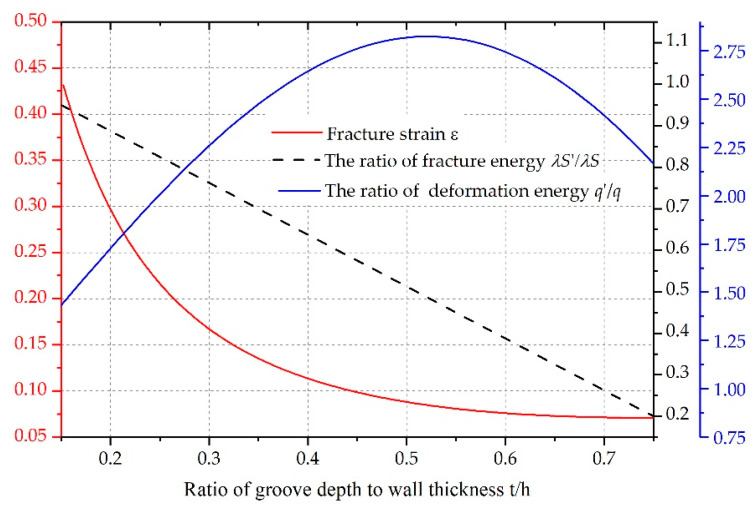
Fracture strain (red line), ratio of elastic energy per unit volume $q^{\prime }/q$ (blue line), and ratio of total fracture energy $\lambda S^{\prime }/\lambda S$ (dashed black line).

Figure 21 illustrates the fracture strain of grooved shells predicted by the proposed model, the ratio of elastic energy per unit volume on grooved shells to that on continuous shells, and the ratio of total fracture energy on grooved shells to that on continuous shells, as a function of *t*/*h*. As *t*/*h* increases, the total fracture energy required to create a new fracture surface decreases linearly, whereas the elastic energy per unit volume exhibits a polynomial increase. In fact, due to the presence of geometric defects, it takes less energy for cracks to penetrate inside the grooved shells than inside continuous shells. Additionally, when subjected to internal explosive loading, the grooves act as stress raisers and induce higher levels of local stress or strains in the adjacent matrix, thereby reserving more elastic energy per unit volume for crack propagation and penetration. Recall that in Equation (8), the total fracture energy is the product of the elastic deformation energy per unit volume and the total volume deformation of the shell, namely, the product of the elastic energy per unit volume and the strain of the shell. Therefore, based on this energy conservation law, the drops in the total fracture energy and the increase in the elastic energy per unit volume may combine to decrease the total volume deformation, thus leading to a polynomial decline in the fracture strain of grooved shells compared to the condition of no grooves on shells.

These findings, though not intuitive, may contribute to a better understanding of the mechanism of geometric defects on fracture promotion.

## 5. Conclusions

In the present study, an existing theoretical model from Ivanov was modified to predict the fracture strain of internally grooved warhead shells by incorporating numerical simulation results that include the effects of groove angle θ, groove depth to thickness ratio *t*/*h*, and shell outer radius to wall thickness ratio *R*_0_/*h*. Considering the fracture promotion effect of geometric defects on shells, we modified Ivanov’s theory by adding the elastic energy liberated per unit volume for crack penetration and reducing the required fracture energy. The proposed model showed good agreement with experimental results and gave a reasonably more accurate estimate compared to the estimate from other formulas.

This study may indicate that the increased elastic energy caused by stress concentration and the reduced fracture energy induced by geometric defects combine to promote the destruction of grooved shells. On the one hand, when the detonation front arrives at the groove tips, a higher level of local stress or strain occurs in the vicinity of the groove, thereby leading to a more increased deformation energy per unit volume inside a grooved shell than inside a continuous shell. On the other hand, the presence of grooves shortens the path the cracks must penetrate, which decreases the total fracture energy compared to the case without grooves present. Note that the total fracture energy is the product of the elastic energy per unit volume and the total deformation or strain amount of the shell. As a result, the increased elastic deformation energy and reduced fracture energy result in a combined decrease in the fracture strain of grooved shells.

Note that the groove root radius is one of the dominant parameters that may affect the strain/stress fields at groove tips and shear band sensibility. However, due to the limitation of the article length and complex nature of groove geometric parameters, we did not take the notch root radius into account. In our theoretical model, we only considered the effects of θ, *t*/*h*, and *R*_0_/*h* on warhead shell fracture. Further work needs to be carried out to study the effects of the groove root radius on shell fragmentation, and we will gradually improve the theoretical model by incorporating the factor of groove root radius. Another limitation of this study is that we do not pay much attention to the effects of stress triaxiality and Lode parameter on warheads disintegration. In fact, the deformation and fracture of materials under warhead explosions and hypervelocity impacts are related to the stress triaxiality and the complex stress state (Lode parameter). The dependence of critical fracture strain on stress triaxiality, the Lode parameter, and the relative crack length (i.e., length of a crack divided by wall thickness) are central issues in dynamic disintegration of grooved shells and should receive considerable attention. However, in this article, we do not go into the details of the material fracture criteria and we pay attention to the change in elastic deformation energy and fracture energy when grooves are machined on shells. We wish to extend this topic in our continued study.

Despite these limitations, the present study contributes to a better understanding of the mechanism whereby geometric defects promote fracturing, and will be of interest to warhead designers who wish to predict the fracture strain of internally grooved shells. Except for internal grooves obtained by machining, controlled fragmentation technologies for manufacturing warheads can also fall into other categories: external grooving, both internal and external grooving, explosive shaping, and electron beam or laser drilling. As a result, we plan to extend the present study to determine the fracture strain of warhead shells with other controlled fragmentation technologies. All of these will be explored based on the present energy conservation method.

## Figures and Tables

**Table 1:** Geometric parameters of the grooved shell used to determine SCFs.

Component	NO.	Outside Radius of Shell (*R*_0_, mm)	Wall Thickness (*h*, mm)	Groove Depth (*t*, mm)	Groove Angle (θ, °)	*R*_0_/*h*	*t*/*h*
1	1	50	6	2.4	45, 50, 55, 60, 65, 70, 75	8.33	0.4
	2	80	8	2.4	45, 50, 55, 60, 65, 70, 75	10	0.3
	3	75	5	3.0	45, 50, 55, 60, 65, 70, 75	15	0.6
2	4	50	6	1.2, 1.8, 2.4, 3.0, 3.6, 4.2	60	8.33	0.2~0.7
	5	80	8	1.6, 2.4, 3.2, 4.0, 4.8, 5.6	75	10	0.2~0.7
	6	75	5	1.6, 2.4, 3.2, 4.0, 4.8, 5.6	60	15	0.2~0.7
3	7	100, 90, 80, 70, 60, 50	6	2.4	60	16.7, 15, 13.3, 11.7, 10, 8.33	0.4
	8	133.3, 120, 106.7, 93.3, 80, 66.6	8	2.4	75	16.7, 15, 13.3, 11.7, 10, 8.33	0.3
	9	83.3, 75, 66.7, 58.3, 50, 41.7	5	3.0	70	16.7, 15, 13.3, 11.7, 10, 8.33	0.7

**Table 2:** Summary of experimental conditions.

Experiment Number.	Explosive Charge	Outer Diameter of Shells (mm)	Wall Thickness *h* (mm)	Groove Depth *t* (mm)	Groove Angle θ (degree)	*t*/*h*	Groove Root Radius (mm)
No. 1	Comp-B	98	6	1.2	60	0.2	0.3
No. 2	Comp-B	98	6	2.4	60	0.4	0.3
No. 3	Comp-B	98	6	3.0	60	0.5	0.3
No. 4	Comp-B	98	6	4.2	60	0.7	0.3

**Table 3:** Summary of experimental results.

Experiment Numbers	*t/h*	*v* (m/s)	$\overset{\cdot }{\varepsilon_{f}}\;(10^{4}\;\times \;s^{-1})$	Theoretical Prediction of Fracture Strain ε				
				AL-Hassani and Johnson [11]	Ivanov [12]	Theoretical Prediction	Experimental Result ^1^	Error ^2^
No. 1	0.2	1472	2.46	0.462	0.398	0.194	0.208	6.7%
No. 2	0.4	1335	2.39	0.405	0.406	0.104	0.115	9.6%
No. 3	0.5	1228	2.25	0.365	0.410	0.086	0.096	10.4%
No. 4	0.7	1125	2.09	0.353	0.426	0.072	0.081	11.1%

^1^ The middle portion of the fragment is selected for wall thickness measurement, and the fracture strain is obtained based on an average fragment thickness. ^2^ Error of theoretical prediction with respect to experimental data.

## Data Availability

Data is contained within the article.
